# Benchmarking Age-Friendly University Practices: AFU Inventory and Campus Climate Survey (ICCS) Study Insights

**DOI:** 10.1093/geroni/igab046.039

**Published:** 2021-12-17

**Authors:** Joann Montepare, Nina Silverstein

## Abstract

The Age-Friendly University (AFU) initiative endorsed by GSA’s Academy for Gerontology in Higher Education (AGHE) provides institutions of higher education with guiding principles for addressing the needs of aging populations. Benchmarks are now needed for assessing age-friendly academic, workplace, and physical campus environments, perceptions of campus constituents, and recommendations for advancing age inclusivity. This symposium will discuss what the AFU Inventory and Campus Climate Survey (ICCS) administered to a national sample of colleges and universities is revealing about the study of age-friendliness in higher education. The sample includes data from over 10,000 faculty, staff, students, and older learners surveyed in 2020-21. Whitbourne will introduce the conceptual model that served as the foundation for the ICCS, with special attention to the need to assess and compare “objective” age-friendly practices with “subjective” perceptions of these practices. Bowen will describe the utility of examining age-friendliness across institutional units with different functions: outreach-engagement, personnel, physical environment, research, services-resources, student affairs, and teaching-learning. Beaulieu will present data demonstrating the importance of assessing perceptions of specific constituent groups including faculty, staff, students, and lifelong learners. Montepare will discuss insights gained about the definition and manifestation of what it means to be ageist, age-friendly, and age-inclusive in higher education. Silverstein will describe strengths and challenges observed across campuses along with recommendations and promising new directions for advancing age inclusivity in higher education.

